# From standard therapies to monoclonal antibodies and immune checkpoint inhibitors – an update for reconstructive surgeons on common oncological cases

**DOI:** 10.3389/fimmu.2024.1276306

**Published:** 2024-04-23

**Authors:** Leonard Knoedler, Lioba Huelsboemer, Katharina Hollmann, Michael Alfertshofer, Konstantin Herfeld, Helia Hosseini, Sam Boroumand, Viola A. Stoegner, Ali-Farid Safi, Markus Perl, Samuel Knoedler, Bohdan Pomahac, Martin Kauke-Navarro

**Affiliations:** ^1^ Department of Plastic, Hand, and Reconstructive Surgery, University Hospital Regensburg, Regensburg, Germany; ^2^ Division of Plastic Surgery, Department of Surgery, Yale New Haven Hospital, Yale School of Medicine, New Haven, CT, United States; ^3^ Department of Pathology, Massachusetts General Hospital, Harvard Medical School, Boston, MA, United States; ^4^ Faculty of Medicine, University of Wuerzbuerg, Wuerzburg, Germany; ^5^ Division of Hand, Plastic and Aesthetic Surgery, Ludwig-Maximilians University Munich, Munich, Germany; ^6^ Department of Internal Medicine III (Oncology and Haematology), University Hospital Regensburg, Regensburg, Germany; ^7^ Leibniz Institute for Immunotherapy, Regensburg, Germany; ^8^ Department of Plastic, Aesthetic, Hand and Reconstructive Surgery, Burn Center, Hannover Medical School, Hannover, Germany; ^9^ Craniologicum, Center for Cranio-Maxillo-Facial Surgery, Bern, Switzerland; ^10^ Faculty of Medicine, University of Bern, Bern, Switzerland

**Keywords:** breast cancer, sarcoma, head and neck cancer, skin cancer, malignant melanoma, monoclonal antibody, immunotherapy, immune checkpoint inhibitors

## Abstract

Malignancies represent a persisting worldwide health burden. Tumor treatment is commonly based on surgical and/or non-surgical therapies. In the recent decade, novel non-surgical treatment strategies involving monoclonal antibodies (mAB) and immune checkpoint inhibitors (ICI) have been successfully incorporated into standard treatment algorithms. Such emerging therapy concepts have demonstrated improved complete remission rates and prolonged progression-free survival compared to conventional chemotherapies. However, the in-toto surgical tumor resection followed by reconstructive surgery oftentimes remains the only curative therapy. Breast cancer (BC), skin cancer (SC), head and neck cancer (HNC), and sarcoma amongst other cancer entities commonly require reconstructive surgery to restore form, aesthetics, and functionality. Understanding the basic principles, strengths, and limitations of mAB and ICI as (neo-) adjuvant therapies and treatment alternatives for resectable or unresectable tumors is paramount for optimized surgical therapy planning. Yet, there is a scarcity of studies that condense the current body of literature on mAB and ICI for BC, SC, HNC, and sarcoma. This knowledge gap may result in suboptimal treatment planning, ultimately impairing patient outcomes. Herein, we aim to summarize the current translational endeavors focusing on mAB and ICI. This line of research may serve as an evidence-based fundament to guide targeted therapy and optimize interdisciplinary anti-cancer strategies.

## 1 Introduction

Cancer persists as a worldwide healthcare burden currently affecting 18 million US Americans (1). Malignant tumors are the second leading cause of death in the US population accounting for 609,360 deaths in 2022 (2). Annual cancer therapy costs amount to more than $200 billion. About one-third of cancer patients report symptoms of reactive depression representing an additional burden beyond the tumor disease (3).

In most solid malignancies, advanced or high-risk cancer commonly warrants a joint approach including local treatment and systematic therapy (4–9). Over the past decades, chemotherapeutics and radiotherapy have been considered the standard (neo-)adjuvant therapy strategies. However, recent scientific advances have proposed novel treatment protocols and broadened the therapeutic arsenal of healthcare providers (10).

The advent of monoclonal antibodies (mAB) (e.g., the anti-human epidermal growth factor receptor 2 (HER-2)/neu antibody trastuzumab) and immune checkpoint inhibitors (ICI) (e.g., programmed cell death protein 1 (PD-1), programmed cell death protein ligand 1 (PD-L1), and cytotoxic T-lymphocyte antigen 4 (CTLA-4) inhibitors) has heralded a paradigm shift in oncology (11, 12). ICI target the immune system evasion mechanism of tumor cells based on self-tolerance-inducing proteins on the cell surface. Common target structures include PD-1 and its ligand PD-L1, both inhibitors of antitumoral T cells (13, 14). The combined positive score (CPS) is used to judge the level of PD-L1 expression, by evaluating the proportion of potential PD-L1 expression, encompassing both tumor and immune cells, relative to the overall count of living tumor cells (15). The clinical effects of mAB (as targeted therapy) center on the stimulation of different innate immune effector processes, direct antibody-mediated toxicity, and complement activation (16). Specific cell surface antigens and receptors, like, epidermal growth factor receptor (EGFR), and vascular endothelial growth factor receptor (VEGFR), represent established targets for mAB (17). The therapeutic potency and clinical efficacy have led to increased rates of complete remissions and prolonged progression-free survival (PFS) even in advanced neoplasms (18). However, the administration of ICI and mAB can induce various side effects. For instance, ICI have been associated with pneumonitis, hepatitis, and myocarditis, while mAB have been implicated with cytokine release syndrome, pneumonitis, cytopenia, or cardiac failure (19–24).

Knowledge of the efficacy, safety, and drug interactions of these emerging treatments is pivotal to leveraging systemic therapies with local surgical strategies (25). There is a scarcity of research work condensing the scientific literature on mAB and ICI for the surgical readership. This knowledge gap leads to untapped therapy potential impairing patient outcomes and survival rates.

Additionally, current cancer guidelines recommend a close interlocking of cancer resection and reconstructive surgery to restore the functionality and aesthetics of the affected body region. A mounting body of evidence has underscored the clinical impact of reconstructive surgery to support patient rehabilitation and improve quality of life (26–29).

Therefore, we aim to summarize the current body of evidence and clinical trials on mAB, ICI, and other immunotherapies for the most common cancer entities treated by reconstructive surgeons (i.e., head and neck cancer (HNC), breast cancer (BC), skin cancer (SC), and sarcoma). This line of research may help reconstructive surgeons develop a more comprehensive understanding of cancer therapies and optimize the perioperative workflow.

## 2 Head and neck cancer – basic information

HNC describes a heterogeneous and complex group of malignancies, categorized based on their anatomical location in the oral cavity, oropharynx, nasopharynx, hypopharynx, and larynx. The majority of HNC (90%) stems from the epithelial lining and is summarized as HNSCC (30–32). Given the majority of HNSCC within HNC, this review focuses on HNSCC and its current neoadjuvant and adjuvant immunotherapeutic modalities. Worldwide, HNSCC is accounting for more than 700,000 new cases and 300,000 deaths per year, representing the sixth most common cancer (33, 34). The overall incidence of HNSCC is proposed to increase by 30% unitl 2030, both in developed and developing countries (32, 35). Significant epidemiological variations in the incidence of HNSCC include tobacco and alcohol consumption, environmental pollutants, and viral infections, namely human papilloma virus (HPV) and Epstein-barr virus (EBV) infections (31, 34). A substitute biomarker for HPV infection is protein p16, which is upregulated by HPV virus proteins (36–38). The American Cancer Society estimated 54,540 new oral cavity and pharynx cancer cases for both sexes in the US of which about 75% occur in male patients (39). The gender dysbalance might be due to a higher prevalence of behavioural risk factors (e.g., nicotine or alcohol abuse) in males (40). Besides these, genetic syndromes (e.g., Fanconi anemia) may contribute to HNSCC (41).

### 2.1 Standard therapy for head and neck cancer

The neoadjuvant therapy typically involves chemotherapy, with a preference for platinum-based compounds like cisplatin, often used in conjunction with taxanes or 5-fluorouracil (5-FU), and might succeeded by radiotherapy or chemoradiotherapy (which may warrant further surgical management due to side-effects) to further halt the tumor’s progression. For locally advanced HNSCC, the primary approach is commonly surgery followed by adjuvant radiation therapy or definitive simultaneous chemoradiotherapy, with surgery (in an organ-preservation approach) kept as a salvage option (42). As delineated in the National Comprehensive Cancer Network (NCCN) Guidelines, Version 2.2020, salvage surgery can also present a curative opportunity for patients enduring isolated resectable recurrences. Treatment decisions are contingent upon several factors, such as the patient’s prior therapy, location, and the extent of recurrence. The treatment modality for unresectable diseases generally mirrors the strategy deployed for metastatic HNSCC. Yet, patients with unresectable HNSCC may still undergo definitive radiotherapy. For metastatic or widespread recurrent HNSCC, systemic therapy is the mainstay of treatment. This typically involves the use of chemotherapy, with platin derivates, taxanes, and 5-FU among others, utilized individually or in various combinations. In such settings, targeted therapies like the EGFR-inhibitor cetuximab have also proven efficacious (43).

In summary, the standard treatment approach for HNSCC, either applied in a neoadjuvant or adjuvant setting, currently consists of surgery and (radio-)chemotherapy but is associated with considerable morbidity and unfavorable prognoses yielding poor five-year survival rates. A landmark Indian multi-institution study reported a five-year cumulative survival (FCS) of 68% for surgical treatment with radiation for oral cavity cancer and 60% for locally advanced stages. Chemoradiation of irresectable cancer compared to radiation alone showed an improvement of 15% in survival rate for oro- and hypopharyngeal cancers with 40% FCS (44). Resistance to radio- and chemotherapy and relapse have been described as the main factors contributing to low survival rates. Additionally, the genetic variety within HNSCC has impeded the process of identifying specific targets and precision therapies. Therefore, gaining a deeper understanding of the pathophysiology of HNSCC is crucial for the advancement of therapy (30). Recent clinical trials indicate that especially mAB immunotherapy can potentially transition paradigms in the treatment of HNSCC (45).

### 2.2 The role of monoclonal antibodies in head and neck cancer therapy

Early favor was assigned to cetuximab, an EGFR-inhibiting mAB. EGFR is a receptor tyrosine kinase upregulated in many malignancies. Its overexpression or overactivation sets off downstream pro-oncogenic pathways, promoting cell proliferation and inhibiting apoptosis (45, 46).

Initially examined in combination with radiotherapy, additional cetuximab showed longer locoregional control (24.4 months) compared to radiation alone (14.9 months). Furthermore, cetuximab and radiotherapy achieved significantly longer overall survival (49.0 months) than radiotherapy alone (29.3 months). Side effects did not differ significantly in both treatment regimens, with the exception of acneiform rash and infusion reactions (47).

In a subsequent phase III trial, including 891 patients, of concurrent accelerated radiation plus cisplatin in combination with cetuximab or alone for stage III to IV HNSCC, the addition of cetuximab led to more interruptions in radiation therapy and more grade III to IV radiation mucositis but did not improve three-year PFS or overall survival. Notably, patients with p16^+^ oropharyngeal carcinoma demonstrated better three-year PFS and overall survival compared to those with p16^-^ oropharyngeal carcinoma, while tumor EGFR expression did not significantly affect the outcome (48).

However, when examined in combination with chemotherapy in the palliative setting, cetuximab in combination with platinum-based chemotherapy and 5-FU, can prolong the median overall survival from 7.4 months to 10.1 months when applied as a first-line treatment for patients with recurrent or metastatic (R/M) HNSCC (49). Therefore, cetuximab was the first introduced targeted therapy for HNSCC (50). Under special recommendations by the National Comprehensive Cancer Network (NCCN), cetuximab is currently considered to be an option for the treatment of patients with R/M HNSCC alongside cisplatin (45, 51). It can be utilized for both HPV-positive and HPV-negative cases and in an adjuvant, neoadjuvant, or combination setting (52).

Skin toxicities have presented as a major side effect in treatment with cetuximab (53). Therefore, a phase IV study is designed to distinguish between skin reactions caused by radiation and those triggered by cetuximab to assess the incidence and severity of these adverse effects (trial identifier: NCT01553032).

Bevacizumab is humanized mAB targeting VEGF-A. VEGF in general is a stimulator of angiogenesis, a crucial factor for tumor growth and metastasis. Its overexpression has been associated with unfavorable outcomes in HNC (45). Several pro-angiogenic factors besides VEGFR, like EGFR, fibroblast growth factor receptor (FGFR), and platelet-derived growth factor receptor (PDGFR), are upregulated. Therefore, along with bevacizumab, there have been other angiogenesis-inhibiting drugs tested, yielding varying degrees of success (54).

However, till 2019, only bevacizumab has been investigated in phase III clinical trials (54, 55). Still, bevacizumab is not recommended by NCCN due to haematologic and other toxicity effects (56). It has been tested alongside docetaxel, a taxane. Taxanes are a group of chemotherapy drugs, which are a pivotal part of chemotherapeutic regimens, especially in breast, ovarian, non-small cell lung, and head and neck cancer. Acting as microtubule inhibitors by impeding the cell cycle at the G2/M phase, this drug class triggers the activation of cellular apoptosis pathways, thus inhibiting cancer growth (57, 58).

**Figure 1:**
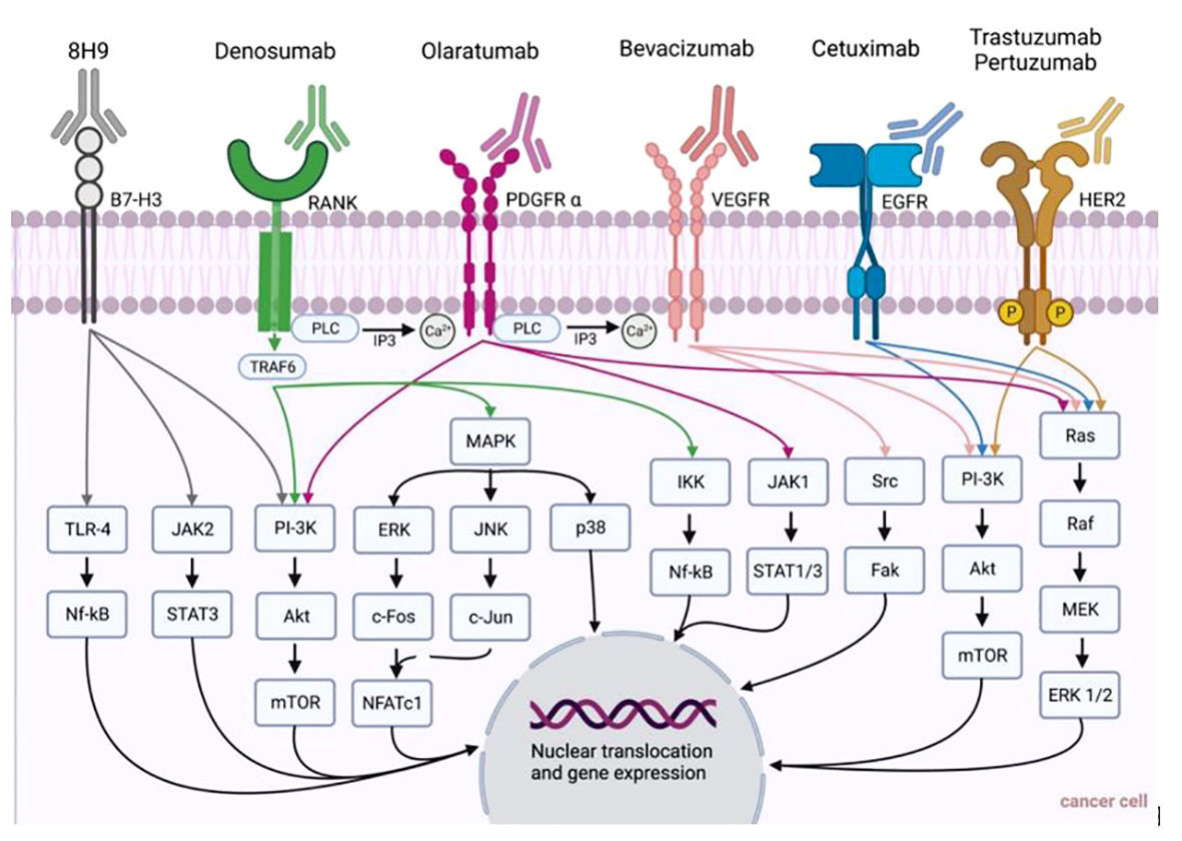
Summary of monoclonal antibodies and their pathways for the treatment of cancer entities presented in this review (i.e., head and neck cancer, breast cancer, malignant melanoma, sarcoma).

A phase II interventional study including 30 patients with stage III or IV HNSCC treated with a combination of bevacizumab, docetaxel, and radiotherapy to determine the treatment-specific time to progression. The three-year PFS was 61.7% with an overall survival of 68.2%. Locoregional recurrence-free survival was 84.5% and distant metastasis-free survival was 80.5%. The most common non-hematologic toxicities were dysphagia, mucositis, and dermatitis. No grade V toxicities were observed, however, more than 80% of patients developed ≥ grade III lymphopenia and two patients suffered from hemorrhage. Still, Yao et al. claimed this regimen as tolerable and effective in HNSCC (59). A phase III randomized trial (E1305) showed improved response rate and PFS with bevacizumab in addition to conventional chemotherapy in the first-line therapy of R/M HNSCC. Overall response rates were 35.5% with bevacizumab alongside chemotherapy and 24.5% with chemotherapy alone. However, there was no significantly improved overall survival, and toxicities were elevated, including a higher rate of treatment-related grade 3 to 5 bleeding events (6.7% vs. 0.5%) and treatment-related deaths (9.3% vs. 3.5%) with added bevacizumab vs. chemotherapy alone. Median overall survival with bevacizumab plus chemotherapy was 12.6 months and 11.0 months with chemotherapy alone. Moreover, at 2, 3, and 4 years, the overall survival rates were 25.2% vs. 18.1%, 16.4% vs. 10.0%, and 11.8% vs. 6.4% for bevacizumab in addition to chemotherapy vs. chemotherapy alone (60) (**Table 1**; **Figure 1**).

**Table 1:** Summary of clinical trials on monoclonal antibodies and immune checkpoint inhibitors for the treatment of the cancer entities presented in this review (i.e., head and neck cancer, breast cancer, malignant melanoma, sarcoma).

Cancer entity	Study/Trial number	Treatment regimen/Antibody	Cancer specification	Drug targets
Monoclonal Antibodies				
Head and neck cancer	NCT01553032 (Phase IV)	**Cetuximab**	Locally advanced (Stage III, IVA or IVB) non-metastatic HNC	**EGFR**
Breast cancer	NCT00411788 (Phase II)	**Trastuzumab** + Rapamycin	positive metastatic breast cancer	**HER-2**
Breast cancer	NCT03765983 (Phase II)	**Trastuzumab** + GDC-0084	positive metastastic breast cancer	**HER-2**
Immune Checkpoint Inhibitors				
Head and neck cancer	NCT03818061 (Phase II)	**Atezolizumab** **+ Bevacizumab**	Advanced/metastatic HNSCC	**PD-L1 + VEGFR**
Head and neck cancer	NCT05187338 (Phase II)	**Ipilimumab + Pembrolizumab + Durvalumab**	Advanced solid Tumors	**CTLA-4 + PD-1 + PD-L1**
Head and neck cancer	NCT03765918 (Phase II)	**Pembrolizumab**	Previously untreated, resectable, locally advanced HNSCC	**PD-1**

### 2.3 Immune checkpoint inhibitors for the treatment of head and neck cancer

In 2016, ICI were introduced for the treatment of HNSCC, when the anti-PD-1 monoclonal antibodies pembrolizumab and nivolumab received approval from the FDA for the treatment of patients with R/M HNSCC. The consent was supported by two landmark trials, stating that treatment with nivolumab led to longer overall survival compared to standard treatment options, and pembrolizumab showed adequate tolerability and clinically relevant antitumor activity in R/M HNSCC (51, 61, 62).

CheckMate 141, the first reported randomized phase III trial of a PD-1 inhibitor in HNSCC, CheckMate 141, the first reported randomized phase III trial of a PD-1 inhibitor in HNSCC, focused on enrolling patients who had experienced disease progession within six months of undergoing platinum-based chemotherapy. Regardless of tumor PD-L1 status, 361 patients were enrolled in this study. Patients were randomized to receive either 3mg/kg nivolumab every 2 weeks or investigator’s choice of weekly systemic standard therapy (methotrexate, weekly docetaxel, or cetuximab. Patients treated with nivolumab exhibited improved median overall survival (7.5 months vs 5.1 months) and a higher overall response rate (13.3% vs. 5.8%) compared to those receiving chemotherapy. At the first interim analysis, the estimated one-year overall survival was notably higher with nivolumab (36% vs. 16,6%). Moreover, 13.1% patients on nivolumab experienced grade 3/4 treatment related adverse events, as opposed to 35.1% of patients on standard therapy. Upon 2-year follow-up, patients who received nivolumab had a median overall survival of 7.7 months, while those who underwent chemotherapy had a median overall survival of 5.1 months. Antibodies blocking PD-1, namely pembrolizumab and nivolumab, have been added to the treatment of HNSCC (15, 25). Further, Gillison et al. analyzed long-term outcomes with nivolumab as a firstline treatment in recurrent or metastatic HNSCCs. In their 2-year randomized, phase III trial, they found that nivolumab (n=50) improved overall survival compared with the investigator’s control therapy (i.e., chemotherapy only (n=26)) from a median of 3.3 months to 7.7 months (63). Wu et al. prospected that more effective cancer immunotherapy can be achieved by using anti-PD-L1 agents in combination with other therapeutics (64). In fact, preclinical evidence from different cancer entities lends supports to therapeutic synergies between chemo- and immunotherapy, primarily due to the activation of innate immune pathways (65, 66).

The palliative first-line therapy standard has fundamentally changed due to the phase III KEYNOTE-048 study, where pembrolizumab alone or in combination with cisplatin, and 5-FU was compared to cetuximab-chemotherapy, in the above-described combination of cetuximab, platinum-based chemotherapy, and 5-FU, in R/M HNSCC. In this study, 301 patients received pembrolizumab alone, 281 pembrolizumab and chemotherapy, and 300 cetuximab and chemotherapy. Both pembrolizumab and pembrolizumab-chemotherapy treatments were compared to cetuximab-chemotherapy. Particularly in tumors with high PD-L1 expression (CPS ≥ 20), pembrolizumab monotherapy extended overall survival to 14.9 months compared to 10.3 months, with a significantly better side-effect profile. Albeit the response rate was lower (23.3% vs. 36.1%) compared to cetuximab-chemotherapy. Overall survival also benefited (given a CPS ≥ 1) at 12.3 months compared to 10.3 months, but 38.9% of this group initially progressed. Overall survival with pembrolizumab in combination with cisplatin, and 5-FU, also significantly extended at CPS ≥ 1, with a similar remission rate, but with a higher side effects rate than pembrolizumab alone, yet comparable to cetuximab-chemotherapy. Patients were followed-up for four years. The median overall survival improved by a minimum of 3.6 months when pembrolizumab was applied either way. In conclusion, first-line pembrolizumab and pembrolizumab-chemotherapy treatment were considered beneficial in R/M HNSCC (67, 68).

Further, PD-1 levels among HNSCC patients infected with HPV have been shown to be elevated (36–38). Additionally, PD-L1 expression is linked to the primary tumor location, postoperative recurrence, survival rate, and PD-1 and p16 expression.

A lower probability for postoperative recurrence was linked to PD-L1^+^/PD-1^+^ (2.5-fold). PD-L1/PD-1 expression, linked to p16 and HPV, in HNSCC patients also led to better overall survival. PD-L1 expression was positively associated with the time of overall survival compared to negative PD-L1 expression (overall survival rate: 82.8% vs 53.5%; overall survival 36.66 months vs. 30.54 months). In conclusion, these findings promote PD-1-targeted immunotherapy for treatment in HNSCC, specifically in HPV-positive cases (36). Therefore, it is paramount to differentiate between HPV-positive versus HPV-negative HNSCC.

Ongoing studies are investigating other potential immune implications and combinations of cancer-related pathways beyond PD-1 or EGFR An active, not recruiting study aims to investigate the clinical and biological effects of the therapy R/M HNSCC with atezolizumab, targeting PD-L1, in combination with bevacizumab, mAB against VEGF (trial identifier: NCT03818061). Of note, atezolizumab in combination with bevacizumab has been tested in a phase II study for patients with unresectable or metastatic mucosal melanoma, demonstrating a median progression-free survival of 8.2 months, along with an objective responste rate of 45.0% (69).

**Figure 2:**
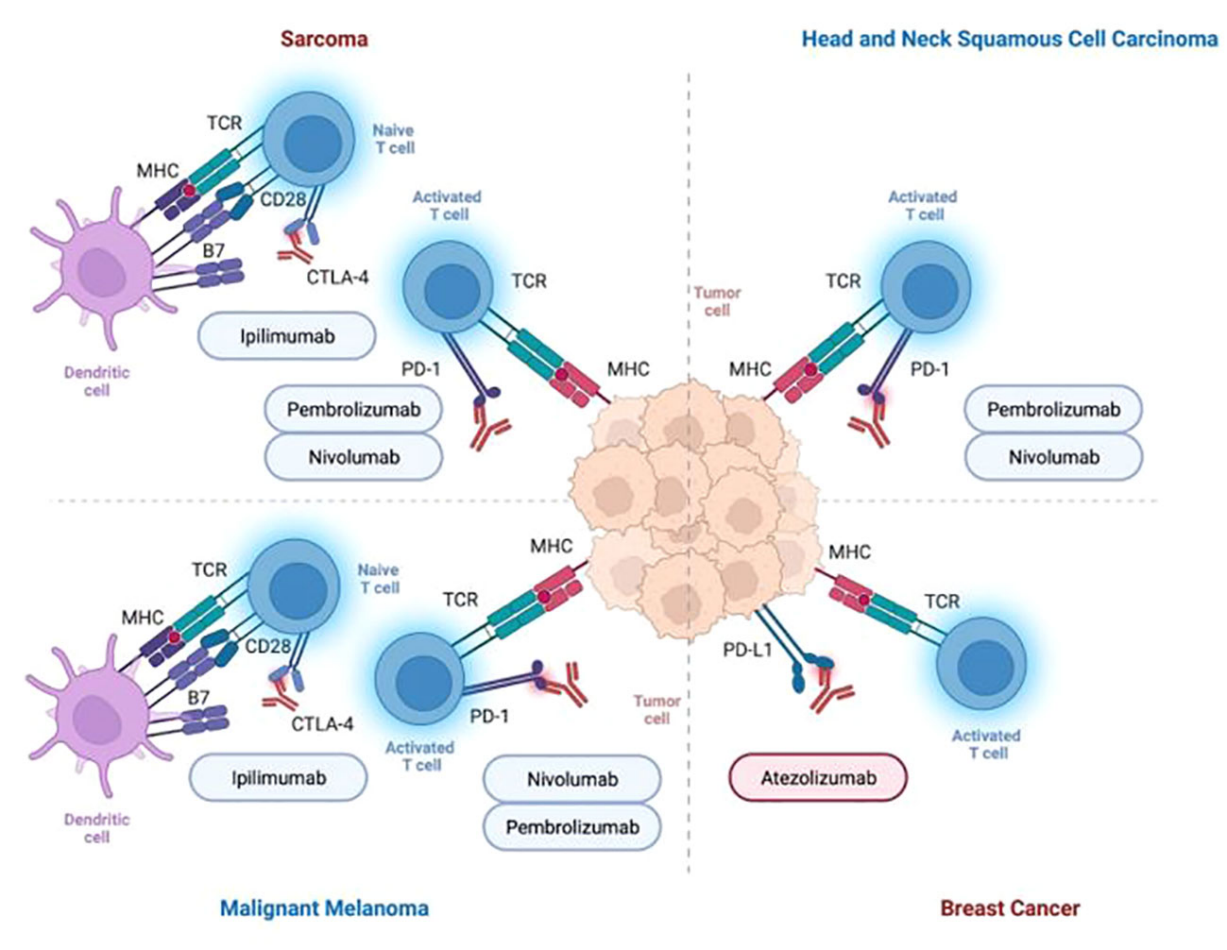
Summary of immune checkpoint inhibitors for the treatment of cancer entities presented in this review (i.e., head and neck cancer, breast cancer, malignant melanoma, sarcoma).

To avoid the above-mentioned ICI treatment failure, pembrolizumab will be used in combination in several studies. For instance, a triple combination of CTLA-4, PD-1, and PDL-1 blocking ICI, ipilimumab, pembrolizumab, and durvalumab, is about to be tested for 100 patients with advanced solid tumors including HNSCC in a recruiting phase I/II trial (trial identifier: 'NCT05187338) (**Table 1** **;** **Figure 2** **).**

### 2.4 Additional therapeutic approaches in head and neck cancer

Tyrosine kinase inhibitors (TKI) work by inhibiting pathways that regulate cell growth, division, and survival but do not belong to the group of mAB and ICI from a pharmacological standpoint (70). Yet, these agents can block multiple angiogenic signaling pathways including those of VEGFR, EGFR, FGFR, and PDGFR. Examples are cabozantinib, axitinib, and lenvatinib (55, 71).

A phase II trial investigated cabozantinib, a multi-TKI inhibiting VEGFR 1-3 among other receptors, in combination with pembrolizumab in the treatment of patients with R/M HNSCC. Median overall survival was 22.3 months with median PFS of 14.6 months. Promising clinical activity and tolerability was concluded, however no correlation between tumor mutational burden (TMB) and clinical outcome was observed, warranting for further investigation for similar combinations for HNSCC (71).

### 2.5 Immunotherapies in head and neck cancer standard therapy – where do we stand?

ICI such as pembrolizumab and nivolumab have been implemented into the treatment algorithm for R/M HNSCC and are an integral part of the most recent American Society of Clinical Oncology (ASCO) Guidelines (72).

For patients with R/M HNSCC, PD-L1 immunohistochemistry and TMB testing are recommended. Pembrolizumab has emerged as a primary treatment option for R/M HNSCC, either paired with platinum/5-FU or used in isolation for patients with a CPS of ≥ 1 (43, 73). In general, PD-L1 CPS of ≥ 1 or TMB of ≥ 10 suggests a positive clinical response to PD-1 inhibitors. Alternatively, based on the performance status, such patients can be administered a combination of pembrolizumab, platinum, and 5-FU.

Interestingly, this therapy approach can be employed for cases with CPS < 1, too. Further, pembrolizumab or nivolumab should be administered to patients with platinum-refractory R/M HNSCC, regardless of CPS status (74). ICI targeting PD-1 should be considered if the tumor has progressed after platinum-based therapy. Radiation therapy is recommended as a therapeutic option concurrently with immunotherapy for palliation or local control in patients with oligometastatic HNSCC. Yet, it is not recommended to be given in order to enhance response to immunotherapy outside of clinical trials. In the specific case of TMB-high R/M HNSCC or PD-L1-positive R/M salivary gland cancer, pembrolizumab is recommended as another therapy option (75).

In conclusion, immunotherapy is the new standard approach for the treatment of R/M HNSCC. Positive results have been achieved in neoadjuvant settings, and combination therapy, including TKI, seems to be a promising strategy but warrants further research and broad clinical studies (76). Currently approaching this concern, the KEYNOTE-689 trial, a phase III randomized, open-label study, is designed to assess pembrolizumab in combination with standard treatment – radiotherapy with or without cisplatin – as both neoadjuvant and adjuvant therapy for patients with previously untreated, resectable, locally advanced HNSCC (trial identifier: NCT03765918).

## 3 Breast cancer – general information

Female BC has taken the lead as the most frequently diagnosed cancer according to global cancer statistics in 2020. Approximately 2.3 million new cases of BC and 680.000 deaths due to BC were estimated worldwide, making it the second leading cause of cancer-related death among women (39, 77).

BC is a generic term referring to a group of heterogeneous malignancies, clinically classified into three primary subtypes based on immunohistochemical markers. Those markers include the status of hormone receptors, either estrogen (ER) or progesterone receptor (PR), and HER-2. Therefore, there are luminal ER^+^ and PR^+^, HER-2^+^, and triple negative BC (TNBC) (78). ER is involved in the main regulation of BC cell growth, PR is considered a significant indicator in carcinogenesis acting as an interdependent partner alongside ER (79). Roughly 15-20% of BC cases exhibit amplification of the HER-2 oncogene, situated on chromosome 17q12. HER-2 is a membrane tyrosine kinase receptor expressed in breast tissue, normal and malignant, indicating pathways for cell proliferation and differentiation. In BC cells, an elevated level of HER-2 is linked to poor prognosis, increased cell proliferation, angiogenesis, and evolution of metastasis (80, 81). Even with adjuvant chemotherapy, the five-year survival rate in metastatic breast cancer remains less than 30% (82, 83). Metastatic recurrence poses a significant challenge for women diagnosed with BC, with 20-30% mortality due to metastatic disease BC (78, 84).

Mutations in the tumor suppressor genes BRCA1 and BRCA2 are responsible for the majority of hereditary BC. Identifying these variants in patients is crucial for determining eligibility for targeted treatment using poly-ADP-ribose polymerase (PARP) inhibitors. Consequently, BRCA1/2 genomic testing is imperative for BC (85). Due to its reliability, accessibility, and simplicity, Sanger DNA sequencing has been recognized as the gold standard for BRCA testing. However, an increase in the application of next-generation sequencing (NGS) technology, which offers recognition of Large Genomic Rearrangements (LGRs) and cost-effectiveness, is observed (86).

### 3.1 Standard therapy for breast cancer – on the pulse of time?

Breast cancer therapy is individualized based on the cancer’s type, stage, and patient’s preference and health factors. Treatment strategies involve surgery, radiation, chemotherapy, hormonal, and targeted therapies.

Neoadjuvant therapy was initially investigated in inoperable, locally advanced BC, but has been integrated into the standard of care, particularly in HER-2^+^ early-stage BC, for 2 cm N0 (according to the TMN classification) or all N^+^ BC subtypes, and TNBC (87). Due to the most recent ASCO guidelines, neoadjuvant therapy is suitable for inflammatory BC patients, and for patients with posttherapeutic tumor residuals. Further, neoadjuvant therapy is applied to reduce the extent of surgery in the breast and axilla and to implement individualized post-neoadjuvant strategies. While TNBC patients with clinically node-positive or T1c disease should receive an anthracycline and taxane regimen prior to surgery, T1aN0 and T1bN0 HER-2^+^ patients have no recommendation for routine neoadjuvant treatment. Postmenopausal patients with hormone receptors-positive, HER-2^-^ BC can be administered hormone therapy to downstage disease preoperatively (88). Moreover, tamoxifen or aromatase inhibitors may be used for hormone receptor-positive tumors. Trastuzumab may be given in HER-2-positive tumors alongside chemotherapy. Primary surgery has been proven to be highly effective. Yet, its clinical impact can be potentiated by including the principles above (89).

Adjuvant therapy after surgery is based on the same pillars as neoadjuvant strategy, namely chemotherapy, radiation therapy, hormonal therapy, and targeted therapy, e.g., anti-HER-2 drugs like trastuzumab for HER-2^+^ tumors (78). While radiotherapy remains crucial in adjuvant BC therapy, ER^+^ tumors are commonly treated with five to ten years of endocrine therapy and/or chemotherapy. Tamoxifen, for example, is a medication classified as a selective ER modulator, which is used for the treatment of BC in both men and women (and as a prophylactic agent for BC risk reduction) (78, 90).

### 3.2 The status quo of monoclonal antibodies in breast cancer therapy

mAB have versatile roles in BC therapy, including directly attacking cancer cells, but also facilitating drug delivery to specific targets, inhibiting cell growth, and blocking immune system evasion. While mAB have proven to be efficient in treating HER-2^+^ BC, their potential remains unexplored for other subtypes, particularly TNBC (79, 91).

Trastuzumab was the first FDA-approved anti-HER-2 mAB in 1998, and remains the first-line therapy for early, advanced, and (in combination therapy) also for metastatic, HER-2^+^ BC (92). By binding to the extracellular domain of HER-2 on tumor cells, it stops the downstream pathways and therefore their proliferation (79). Resistance to trastuzumab-based therapy poses a significant challenge, thus anticipation for enhanced therapeutic outcomes lies within investigating drug combinations that target multiple relevant pathways. Interestingly, trastuzumab is already applied in combination with other agents, e.g. anthracyclines and taxanes, in BC. For metastatic BC, many other drugs are trialed besides trastuzumab (80). Further examples of FDA-approved mAB for BC treatment are pertuzumab, another anti-HER-2 mAB, and bevacizumab. Bevacizumab targets VEGF, therefore acting as an anti-angiogenic drug. Erinjeri et al. retrospectively analyzed 1,108 port placements in patients who were treated with bevacizumab. Ports had to be removed in 120 patients of which 11 were BC patients. Following port removal one BC patient presented with wound healing disorders. Overall, patients with an interval between bevacizumab therapy and surgery of less than 14 days demonstrated a significantly higher risk of wound healing disorders (93). This finding can be due to the half-life of bevacizumab which may vary between 11 and 50 days. The GeparQuinto study by Gerber et al. included 127 study sites and looked into outcomes following BC surgery. The authors found no overall rise in surgical complications when bevacizumab was administered concurrently with neoadjuvant anthracycline- and taxane-based chemotherapy for early or locally advanced BC. Overall, 38.1% of all patients included in the study suffered surgical complications, such as bleeding, necrosis, wound infections, and abscesses. In the group of patients, who were treated without bevacizumab, surgical complications occurred in 10.9% vs. 15.0% when treated with bevacizumab showing no statistical significance. Yet, within specific patient subsets, such as those undergoing breast-conserving surgery or requiring repeated surgical intervention to attain clear margins, the incidence of complications significantly increased among those treated with bevacizumab. Among patients undergoing breast-conserving surgery (N=53), surgical complications occurred in 7.3% following treatment without bevacizumab, and in 13.4% following treatment with bevacizumab (94). Interestingly, to date, no trials correlated ICI in BC therapy with an increased risk of lymphedema, a common complication following breast cancer resection, especially when combined with lymph node dissection (95). Golshan et al. investigated the neoadjuvant administration of bevacizumab in TNBC. While 28 patients received single-agent cisplatin prior to definitive surgery, 51 patients were administered neoadjuvant cisplatin plus bevacizumab preoperative. The authors reported that the use of expanders or implants might be problematic for patients treated with bevacizumab with a trend towards more wound-related events in this group despite not yielding significant differences to the cisplatin-only group. Yet, they emphasized that future research was warranted to deduce concrete recommendations (**Supplementary Material S1**).

### 3.3 What do the guidelines say? – immune checkpoint inhibitors in the standard therapy for breast cancer

Immunotherapy has emerged as a promising lifeline for TNBC patients. It is, therefore, recommended, that all patients eligible for immunotherapy treatment undergo tumor tissue PD-L1 testing, regardless of their prior immunotherapy in adjuvant or neoadjuvant settings.

Current immunotherapies regimens are recommended to include anthracyclines and a taxane, with or without carboplatin. Adjuvant immunotherapy should be balanced carefully, especially regarding potential toxicities. Neoadjuvant pembrolizumab and atezolizumab are recommended based on improved pathological complete response rates, regardless of PD-L1 status. Patients with inoperable advanced or metastatic TNBC should undergo tumor tissue testing for PD-L1 and comprehensive genomic profiling, including TMB and MSI tests.

Pembrolizumab alongside chemotherapy, is beneficial for patients with tumors expressing PD-L1 with a CPS of ≥ 10. It is recommended as a first-line treatment when combined with nab-paclitaxel, or a carboplatin and gemcitabine combination. However, it is also still only recommended when PD-L1 expression has a CPS of ≥ 10. High-risk early-stage TNBC patients can benefit from pembrolizumab with chemotherapy before and after surgery.

Further studies are expected to enhance treatment for early-stage TNBC and other subtypes. Future research may also further explore immunotherapy’s impact on hormone receptor-positive and -negative subtypes, and ideal chemotherapy combinations involving ICI (96).

## 4 Skin cancer – from clinical classifications to genetic profiling

SC is the predominant form of cancer worldwide, accounting for a variety of malignancies. It is generally categorized as non melanoma skin cancer (NMSC), including basal cell carcinoma (BCC) and squamous cell carcinoma (SCC), or as malignant melanoma (MM) arising from the malignant transformation of melanocytes (i.e., melanin-producing cells in the basal layer of the epidermis). Of note, melanocytes originate from the neural crest, indicating that MM can be found in any location, where neural crest cells migrate such as the skin, but also the gastrointestinal tract, the brain or the eye (97, 98). Due to significantly higher mortality rates in MM compared to other SC, this review will focus on MM (97, 98). Of note, immunotherapies (especially ICI) have been shown to be effective therapy agents in NMSC (99).

Multiple epidemiological studies have provided evidence of rising incidence in MM over the past decades (98, 100–102). Global cancer statistics in 2020 reported 325,000 new cases and 57,000 new deaths for MM versus 6.3 million cases and 56,000 deaths for NMSC, underlying the high mortality of MM disease (32). MM commonly affects younger patients compared to other solid tumors and is more common for White people than Black persons or Asians (97, 103). MM development involves multiple factors arising from an interaction between endogenous factors, like genetic predisposition, and exogenous factors. UV solar radiation exposure is the main risk factor for cutaneous malignancies (100, 104). The increased occurrence of UV-induced cytidine to thymidine transitions is associated with a significantly higher mutation rate in MM than in other solid tumors (105, 106). Increased sensitivity to UV radiation, especially in white patients increases the risk of developing MM as pigmentation and melanin are crucial for shielding melanocytes and keratinocytes from UV lights. That is why individuals with lighter skin (i.e., phototypes I and II) face a greater risk for SC (107). Family history is another pivotal risk factor for MM development, while mutations in the tumor suppressor gene cyclin-dependent kinase inhibitor (CDKN2A) are the most common ones responsible for hereditary MM (108, 109). Somatic driver mutations for melanoma include, for example, B rapidly accelerated fibrosarcoma (BRAF), RAS, CDKN2A, neurofibromatosis type 1 (NF1), and phosphatase and tensin homolog gene (PTEN) (110, 111).

### 4.1 Standard treatment approach for malignant melanoma

For local MM therapy, the primary treatment is surgical removal, showing high survival rates. However, survival rates decrease significantly after metastasis (112, 113). Before the advent of immune checkpoint inhibitors, the five-year relative survival rate for patients with stage IV, metastatic MM was around 10%, depending on the location and metastasis burden. For comparison, the five-year relative survival rate for MM stage 0 (i.e., melanoma in situ) is 97% (97). Beyond surgery, immunotherapy plays a key role in standard therapy for MM (Please see “From Bench to Beside – Immunotherapy as Standard Therapy for Malignant Melanomas). Applying immunotherapy in MM treatment, improved survival rates. The five-year overall survival in patients with advanced melanoma was 52% when receiving nivolumab and ipilimumab, and 44% when receiving nivolumab alone (114).

### 4.2 Monoclonal antibodies and immune checkpoint inhibitors for malignant melanoma – effective and targeted therapies?

#### 4.2.1 Monoclonal antibodies for malignant melanoma

Relatlimab, a mAB targeting LAG-3, plus nivolumab, a PD-1-targeting mAB, have been approved by the FDA as a first-line treatment for advanced-stage melanoma (115). While inhibition of PD-1, and PD-L1 mostly (not exclusively) affects CD8^+^ T cells, inhibition of LAG-3 is proposed to affect multiple immune cell subtypes, since LAG-3 is found to be more broadly expressed on various cell lines (116). To investigate relatlimab’s specific cellular impact on the immune system, Huuhtanen et al. performed a phase I single-cell characterization trial. The authors evaluated relatlimab plus nivolumab, stating that the combination of those drugs, inhibiting LAG-3 and PD-1 affected not only CD8^+^ T cells but also natural killer cells (NK cells) and regulatory T cells (T_regs_). Adaptive NK cells (i.e., specialized NK cells carrying the potential for immunological memory) experienced an enhancement in responders and transcriptomic changes, while the number of T_regs_ increased. Interestingly, transcriptome analysis showed that the metabolic activity of T_regs_ decreased. Furthermore, the clonality of T cell receptors, as well as the cytotoxic and NK cell-like activity of their enlarging CD8^+^ T cell clones increased in therapy-responders (117) (**Table 1**; **Figure 1**).

Molecular and cellular factors as suitable targets for checkpoint inhibitors in MM have been identified, including PD-L1, major histocompatibility complex (MHC-I), TMB and BRAF mutation, and T cell infiltration. Additionally, MITF, a transcription factor involved in the development and survival of melanocytes, is proposed to have a mechanistic relevance in MM treatment.

Primary targets in melanoma treatment are PD-1, PD-L1, and CTLA-4. Pembrolizumab and nivolumab target PD-1 to enhance the immune response against melanoma cells, whereas ipilimumab binds to CTLA-4 to support the immune response. The efficacy of immune checkpoint inhibition relies on factors involved in the immune balance with melanocyte survival. PD-L1 expression is emphasized as one key element, intricately interwoven with other molecular and cellular players like MHC expression, and mutational load (112, 118). Regardless of the BRAF mutational status, immunotherapies have shown promising efficacy (119). The human monoclonal IgG1 antibody Ipilimumab received its approval for treating advanced or unresectable MM based on survival benefits for patients with advanced MM (120–123).

In a phase III trial, including 676 patients with pretreated metastatic, unresectable stage III or IV melanoma, ipilimumab, alone or in combination with glycoprotein 100 peptide vaccine (gp100), demonstrated enhanced overall survival compared to gp100 alone. The median overall survival for ipilimumab alone was 10.1 months, combined with gp100 10.0 months, and for gp100 alone 6.4 months. Patients experienced severe therapy-induced immune-related adverse events, most frequently affecting the skin and gastrointestinal tract (i.e., diarrhea, nausea, pruritus), which can be treatment-limiting (120). Ipilimumab was followed by the approval of nivolumab and pembrolizumab (i.e., PD-1 inhibitors) for the therapy of advanced or metastatic melanoma among other tumors. A combination of ipilimumab and nivolumab has been established for the treatment of patients with BRAF wild-type metastatic or unresectable MM (122, 124).

The application of nivolumab, in combination with ipilimumab or by itself, has been shown to be an effective treatment for patients with advanced melanoma. A phase III clinical trial reported a median overall survival rate of more than 60.0 months for patients treated with nivolumab and ipilimumab, 36.9 for patients treated with only nivolumab, and 19.9 months for patients treated with only ipilimumab. The five-year overall survival for patients treated with both agents was 52%, versus 44% with nivolumab, and 26% with ipilimumab. Additionally, quality of life was not permanently impaired (114). A still ongoing multicenter, multi-cohort, open-label phase Ib/II trial was designed as a first-in-human study to investigate the safety, tolerability, and efficiency of a combination of bi-specific antibody applications, targeting different T-cell co-inhibitory receptors for patients with advanced or metastatic melanoma prior immune checkpoint inhibitor therapy. XmAb2284, CTLA-4 X LAG3 or bavunalimab, targeting both CTLA-4 and LAG-3, and XmAb23104, also known as PD1 X ICOS or XmAb104, targeting PD-1 and ICOS (CD278), a costimulatory molecule for T-cell proliferation and cytokine secretion, are applied intravenously (125) (**Table 1**; **Figure 2**).

#### 4.2.2 Immune checkpoint inhibitors for malignant melanoma

T cell immunoglobulin and mucin domain 3 (TIM-3) and T cell immunoreceptor with Ig and ITIM domains (TIGIT) are under investigation as the next wave of co-inhibitory receptor targets. They showcase distinctive functionalities, particularly in regulating the immune system in tissue sites, while being negative regulators of T-cell function like PD-1 and CTLA-4 at the same time. ICI brought about a significant revolution in the treatment of MM, but novel combination therapies are explored to improve response rates (117, 123, 126).

### 4.3 Treatment approaches for malignant melanoma - beyond monoclonal antibodies and immune checkpoint inhibitors

In this context, vemurafenib and dabrafenib, selective mutant BRAF inhibitors, were approved for patients with metastatic MM and BRAFV600 mutations (127, 128). Targeted therapy includes BRAF and mitogen-activated protein kinase kinase (MEK) inhibitors like vemurafenib, dabrafenib, and trametinib. BRAF, a serine/threonine protein kinase activating the mitogen-activated protein kinase (MAPK) and downstream MEK and ERK signaling pathways, plays a crucial role in tumorigenesis (129, 130). The MEK pathway is promoted by BRAF mutations and might be responsible for melanoma cell proliferation (128, 129, 131, 132). More than 50% of MM cases contain activating BRAF mutations, with a substitution of valine to glutamine in codon 600 (V600E) being the most common BRAF mutation (128, 133).

Clinical phase I and II trials suggested that vemurafenib, also known as PLX4032, represented an effective treatment yielding response rates of more than 50% in MM patients with BRAF V600E mutations (134–137). Chapman et al. investigated the efficacy of vemurafenib compared to dacarbazine, the only chemotherapeutic drug approved for metastatic MM therapy, in a phase III randomized clinical trial including 675 patients with untreated, metastatic MM carrying the BRAF V600E mutation. Vemurafenib showed enhanced overall survival with 84% at six months compared to 64% at six months for dacarbazine use, and improved PFS (135). Still, resistance to BRAF inhibitors over time was shown, most likely due to MAPK-driven acquired resistance. Combining both MEK and mutant BRAF kinase was proposed to delay resistance mechanisms (128). Trametinib is a selective, allosteric MEK1/MEK2 inhibitor, also known as GSL1120212, which was recently investigated in a phase II clinical trial designed to determine the response rate for patients with metastatic BRAF mutant melanoma. It showed good patient tolerability and significant clinical tumor reduction in patients who were not treated with BRAF inhibitors before, but poor response rates in patients that had been previously treated with a BRAF inhibitor. Kim et al., therefore, stated that these resistance mechanisms against BRAF inhibitors were likely to pass resistance on to MEK inhibitor monotherapy, warranting further investigations (130, 138, 139).

### 4.4 From bench to beside – immunotherapy as standard therapy for malignant melanomas

While surgery is still the mainstay of treatment, the advent of immunotherapy, specifically the use of the ICI nivolumab and ipilimumab in advanced MM, has revolutionized the therapy of patients with MM (114, 140). In a neoadjuvant setting, an emerging approach for high-risk melanoma may include immunotherapy and agents like ipilimumab, nivolumab, and pembrolizumab, and targeted therapy for melanomas with BRAF mutations.

Adjuvant therapy, aiming to reduce recurrence, also relies on immunotherapy and targeted therapy. The individual treatment approach depends on various factors, like tumor stage, metastasis, and BRAF mutation status. According to the ASCO Guidelines for melanoma treatment, nivolumab and pembrolizumab should be considered for patients with resected stage IIIA/B/C/D BRAF wild-type cutaneous melanoma. These drugs, or a dabrafenib-trametinib combination should be offered for BRAF-mutant tumor patients. No definitive guidance for neoadjuvant cutaneous melanoma treatment has been provided as of 2023. For unresectable or metastatic cases, BRAF wild-type patients should be administered a combination of ipilimumab and nivolumab, nivolumab alone, or pembrolizumab treatment. BRAF-mutant patients can also receive BRAF/MEK inhibitors (e.g., dabrafenib or trametinib). The same treatment strategies are recommended for patients with mucosal melanoma (141, 142).

## 5 Sarcoma – prevalence, incidence, and risk factors

Broadly speaking, sarcomas manifest as either soft-tissue sarcomas (STS) or bone sarcomas (BS), while STS occur with a four to five times higher prevalence than BS (143). STS encompass a range of mesenchymal malignancies originating from muscles, adipose tissue, tendons, and vasculature structures. They predominantly occur in the extremities (143–145). Among children and adolescents, rhabdomyosarcoma (RMS) and synovial sarcoma are the most frequent subtypes, whereas liposarcoma (LS) and leiomyosarcoma (LMS) are the most common STS observed in adults. Based on prevalence rates, this review will focus on RMS, LS, and LMS (143, 144). RMS is categorized into distinct clinical subtypes based on histopathological features including embryonal RMS (ERMS), alveolar RMS (ARMS), pleomorphic RMS, and spindle cell/sclerosing RMS (ssRMS). ERMS and ARMS are the most common subtypes, whereas the other subtypes are considered rare. PRMS stands out due to its non-responsiveness to chemotherapy, unlike ERMS and ARMS. It is often treated with radiation therapy and wide excision (146, 147).

For STS, 13,400 new cancer cases, and 5,140 new deaths in 2023 were estimated for the United States of America (39). STS can be attributed to environmental and/or genetic risk factors (148–150). Germline mutations in the tumor suppressor gene TP53 are accountable for LFS and can contribute to the early onset of sarcomas and other tumors by causing genomic instability (149–152). Even though STS show a relatively low incidence, STS represents an aggressive oncological disease leading to high mortality rates (143). Over the past two decades, no substantial change in the overall and one-year mortality rates among various histological subtypes has been observed.

### 5.1 Standard treatment approach in sarcoma therapy

Surgical resection is the backbone of sarcoma therapy aiming for complete resection with negative margins (i.e., R0), but additional drug therapy is commonly indicated to avoid recurrence and enable resection (144, 153).

RMS is commonly treated with a multimodal approach including chemotherapy, surgical resection, and/or radiation therapy. Localized RMS is curable for most patients. Chemotherapy includes drugs like vincristine, actinomycin D, and cyclophosphamide. Surgery aims for complete tumor removal, with radiation therapy used for unresectable tumors, or if there’s a high risk of local recurrence (154).

Radiation therapy can be employed both in a neoadjuvant and adjuvant approach to impair local recurrence. Anthracycline-based chemotherapy approaches remain the standard initial therapy for LS, both resectable and unresectable. Doxorubicin is applied either as a single agent or in combination with ifosfamide (155, 156). It is important to mention, that treatment option for LS rely on the tumor type and stage. LS can be differentiated into several subtypes, including well-differentiated liposarcoma (WDLPS), dedifferentiated liposarcoma (DDLPS), pleomorphic liposarcoma, and myxoid/round-cell liposarcoma (MRCLS). Those subtypes respond differently to systemic therapies. The most frequent subtypes WDLPS and DDLPS remain challenging to treat due to their low response rate to standard chemotherapy. However, MRCLS, for example, shows higher sensitivity to doxorubicin-based regimens (157).

Surgery is often applied in the treatment of localized LMS, also approaching for R0 resection. For unresectable or metastatic cases, doxorubicin is the most commonly used first-line agent in chemotherapy. For some patients, radiation therapy may be beneficial as an adjuvant therapy strategy, or in palliative cases (158).

However, even with combined (neo-)adjuvant treatment strategies, approximately 50% of treated patients still show relapse episodes. For metastatic and refractory sarcomas, the median survival rate remains merely 12 to 18 months (159, 160). Consequently, novel therapeutic strategies, notably antibody-based therapies are warranted to advance sarcoma treatment and improve patient prognosis (161).

### 5.2 Monoclonal antibodies in sarcoma therapy – fact or fiction?

Olaratumab, a human mAB targeting PDGFR-α, showed enhanced overall survival in phase II trials, when applied in combination with the chemotherapeutic agent doxorubicin, compared to only doxorubicin, for patients with advanced or R/M STS (162, 163). Thus, it got approved by the FDA, but recently has been taken off the market due to underwhelming findings in phase III studies. Tap et al. stated no significant benefit in overall survival by adding olaratumab to doxorubicin in a double-blinded, randomized trial, including 509 patients from 110 sites in 25 countries (162, 164, 165).

A murine IgG1 mAB called 8H9 targets a distinctive cell surface tumor antigen found in neuroectodermal, epithelial, and mesenchymal tumors, including RMS. More precisely, 8H9 targets B7-H3 (also referred to as CD276) by binding to a region that is crucial for the immunologic function of this immune checkpoint and immunoregulatory molecule. Of note, B7-H3 displays elevated expression in tumor tissues versus suppressed expression in normal tissues. Thus, it has been discussed to carry untapped potential as a target for cancer immunotherapy (166, 167). Modak et al. reported *in vitro* characterization of radiolabeled 8H9 and its potential application *in vivo* with subcutaneous human RMS, stating that the utilization of radiolabeled 8H9 showed effective targeting of RMS xenografts and holds promising potential for potentiating radioimmunotherapy (168). Yet, further studies are warranted to corroborate its clinical impact (**Table 1**; **Figure 1**).

### 5.3 Immunotherapeutic and epigenetic options for sarcoma patients

#### 5.3.1 Monoclonal antibodies for sarcoma patients

Pembrolizumab, nivolumab, and ipilimumab have been investigated for their application in sarcoma treatment (169). A phase I/II study of pembrolizumab and doxorubicin for the treatment of unresectable R/M sarcoma did not reach its primary endpoint (i.e., objective response rate) but demonstrated a notable improvement in PFS when compared to conventional treatment protocols. Patients who received pembrolizumab and doxorubicin showed a median PFS of 8.1 months compared to single doxorubicin with a median PFS of under five months (170).

Zhou et al. proposed combining ipilimumab and nivolumab as an efficient and properly tolerated treatment option for advanced STS (171). Chen et al. reviewed 150 patients with untreated PD-L1 positive R/M STS, who have been therapied with nivolumab, ipilimumab, or nivolumab alone. An improved survival rate of 12.2 months compared to 9.2 months was reached when patients received a combination of both drugs compared to nivolumab alone. However, the application of both drugs showed less tolerability compared to nivolumab alone (172) (**Table 1**; **Figure 2**).

#### 5.3.2 Immune checkpoint inhibitors and epigenetic options for sarcoma patients

Undifferentiated pleomorphic sarcoma often presents with a notably dense immune infiltration. ICI targeting PD-1, PD-L1, and CTLA-4 have shown efficacy, with response rates varying between 20% and 40%. A recent phase II trial of neoadjuvant ICI with concurrent radiotherapy (n=10), exhibited a 90% pathologic response rate for nivolumab/ipilimumab in patients with extremity/truncal undifferentiated pleomorphic sarcoma. Adverse effects observed in this study were in line with the known safety profiles of nivolumab and the combination nivolumab/ipilimumab. Postoperative complications, including one anastomotic leak, anemia, and one wound infection also were consistent with expectations. Stating there is no validated cutoff for pathologic response in STS, they used 30% hyalinization as the optimal cutoff for pathologic response with receiver-operating curves based on landmark analysis of early relapse 52 weeks after surgery. A minimum of 30% hyalinization was found in 90% of patients with undifferentiated pleomorphic sarcoma. Also, overall survival at 24 months was 90% in patients with undifferentiated pleomorphic sarcoma (173, 174).

Carcinogenesis extends beyond genetic alterations and encompasses significant involvement of epigenetic mechanisms, especially histone modifications like acetylation, which is managed by histone deacetylase (HDAC). Inhibition of HDAC has been shown to induce apoptosis, differentiation, and cell cycle arrest while reducing angiogenesis and modulating immune response for cancer cells. HDAC inhibitors are tested in several studies for various cancer types, like lymphoma, or BC (175).

An interaction between HDAC inhibitors and the multi-kinase inhibitor pazopanib, both *in vitro* and *in vivo*, has been recently evidenced to induce apoptosis in sarcoma cells and decrease tumor growth (175–177). Pazopanib is a multi-TKI, with targets including VEGFR 1-3, and PDGFR-α, and -β, already approved for the treatment of advanced renal cell carcinoma in the European Union and US (178). Axitinib is another TKI, that has been demonstrated to selectively inhibit VEGFR 1-3 and interact with HDAC inhibitors, ultimately leading to the apoptosis of sarcoma tumor cells *in vivo* and *in vitro* (176, 179). Axitinib was examined in combination with pembrolizumab in patients suffering from advanced sarcomas in a phase II trial, stating a 3-month PFS of 65.6% for eligible patients and 72.7% for patients with alveolar soft-part sarcoma. The most frequent grade 3/4 treatment-related adverse events encompassed hypertension and autoimmune toxicities, both in 15% of 33 patients enrolled. Additionally, the authors of the study reported serious treatment-related adverse events such as autoimmune colitis, transaminitis, pneumothorax, and hemoptysis in 21% of patients. Thus, Wilky et al. stated that the combination of axitinib and pembrolizumab showed promising antitumoral activity and manageable toxicity in patients with advanced sarcomas. However, further investigations are warranted to pinpoint this treatment combination’s clinical and mechanistic effects (180).

### 5.4 Immunotherapies in sarcoma patients – the most recent guideline updates

Atezolizumab is recommended for unresectable or metastatic alveolar soft-part sarcoma. Pembrolizumab is not specifically FDA-approved for STS, but has been granted approval for specific patients with advanced sarcoma showing high MSI, DNA mismatch repair deficiency (dMMR), or high TMB. Dostarlimab, targeting the PD-1/PD-L1 pathway is approved for patients with advanced sarcoma exhibiting dMMR. Denosumab, a mAB targeting the RANKL pathway, is approved for certain subgroups of bone cancer patients (181, 182).

## 6 Discussion

Tumor therapy is a dynamic field and this work shows, it is crucial to work within a multidisciplinary team. Recent advancements have paved the way toward promising treatments that improve complete remission rates and prolong PFS. The knowledge of the strengths and limitations of novel mAB and ICI is paramount to providing effective therapy concepts and leveraging surgical and non-surgical therapies. Thus, reconstructive surgeons should be updated on the latest oncological therapy trends and further explore potential links between mAB/ICI administration and surgical outcomes.

Overall, future clinical trials are warranted to explore further the potential of mAB and ICI in the presented cancer entities. There is a lack of ongoing clinical trials in skin cancer and sarcoma (**Table 1**).

Currently, scrutinized target structures for ICI include PD-1 and its ligand PD-L1, which are both inhibitors of antitumoral T cells. Targets of mAB include EGFR and VEGFR, and HER-2. EGFR and VEGFR are growth-factor receptors, and HER-2 is a membrane tyrosine kinase receptor expressed in breast tissue, an oncogene in BC (13, 14, 17, 80, 81).

Beyond those structures, more targets show potential. Anti-RANKL mAB therapy shows potential in BRCA1-mutational driven BC and is approved for some types of bone cancer (183) (181, 182). In BC, CD4/6 inhibitors are explored - looking at the crucial role of the cell cycle in tumor development (184–188). CTLA-4 is one of the main targets in mAB therapy in MM, but more structures are scrutinized as promising targets like LAG-3 (117). For ICI in MM, TIM-3, and TIGIT are currently under investigation as coinhibitory-receptor targets (126). Beyond mAb and ICI, BRAF inhibitors have been investigated in MM treatment (138). mAB for sarcoma therapy focus on PDGFR-α and B7-H3, while ICI involve HDAC-inhibitors. Further, the application of multi-TKIs targeting VEGFR 1-3, and PDGFR-α, and -β, is under current investigation for sarcoma therapy (169).

This review condenses the current body of evidence on mAB and ICI in HNC, SC, BC, and sarcoma therapy. Ultimately, this line of research goes beyond the scalpel and may serve as an evidence-based fundament to optimize the therapeutic efficacy of surgical and non-surgical treatments.

## Author contributions

LK: Writing – original draft, Writing – review & editing. LH: Writing – original draft, Writing – review & editing. KaH: Writing – review & editing, Writing – original draft. MA: Writing – review & editing, Writing – original draft. KoH: Writing – review & editing, Writing – original draft. HH: Writing – review & editing, Writing – original draft. SB: Writing – original draft, Writing – review & editing. VS: Writing – original draft, Writing – review & editing. AS: Writing – original draft, Writing – review & editing. MP: Writing – original draft, Writing – review & editing. SK: Writing – original draft, Writing – review & editing. BP: Writing – review & editing. MK: Writing – review & editing.

## Glossary

**Table:**

5-FU	(5-)fluorouracil
ARMS	alveolar rhabdomyosarcoma
BAP1	BRAC1 associated protein
BAP1-TPDS	BAP1 tumor predispositions syndrome
BC	breast cancer
BCC	basal cell carcinoma
BRCA1/2	breast cancer gene 1/2
BRAF	v-raf murine sarcoma viral oncogene homolog B1
BS	bone sarcomas
CDK	cyclin-dependent kinase
CDKN2A	cyclin-dependent kinase inhibitor
CPS	Combined Positive Score
CTLA-4	cytotoxic T-lymphocyte antigen 4
CXCR4	CXC chemokine receptor type 4
DDLPS	dedifferentiated liposarcoma
dMMR	DNA mismatch repair deficiency
EBV	epstein-barr virus, human herpesvirus 4
EGFR	epidermal growth factor receptor
ER	estrogen receptor
ERK	extracellular signal-regulated kinase
ERMS	embryonal rhabdomyosarcoma
FCS	five-year cumulative survival
FDA	United States Food and Drug Administration
FGFR	fibroblast growth factor receptor
HDAC	histon deacetylase
HER-2	human epidermal growth factor receptor 2
HNC	head and neck cancer
HNSCC	head and neck squamous cell carcinoma
HPV	human papilloma virus
ICI	immune checkpoint inhibitors
IgG4	humanized mononuclear immunoglobulin G4
LAG-3	lymphocyte activation gene 3
LFS	Li-Fraumeni syndrome
LMS	Leiomyosarcoma
LS	Liposarcoma
mAB	monoclonal antibodies
MAPK	mitogen-activated protein kinase
MEK	mitogen-activated protein kinase kinase
MHC-I	major histocompatibility complex I
MITF	microphthalmia-associated transcription factor
MM	malignant melanoma
MRCLS	myxoid/round-cell liposarcoma
MSI	microsatellite instability
mTOR	mammalian target of rapamycin
NF1	neurofibromatosis type 1
NK cells	natural killer cells
PALB2	partner and localizer of BRCA2 gene
PARP	poly-ADP-ribose polymerase
PD-1	programmed cell death protein 1
PD-L1	programmed cell death protein ligand 1
PDGFR	platelet-derived growth factor receptor
PFS	progession-free survival
PI3K	phosphatidylinositol-3-kinase
PI3KCA	phosphatidylinositol-4,5-biphosphaste 3-kinase catalytic subunit alpha
PR	progesterone receptor
PTEN	phosphate and tensin homolog gene
R/M	recurrent or metastatic
RAC1	ras-related C3 botulinum toxin substrate 1
RANK	receptor activator of nuclear factor kappa-B
RANKL	RANK ligand
RMS	Rhabdomyosarcoma
SC	skin cancer
SCC	squamous cell carcinoma
ssRMS	spinde cell/sclerosing rhabdomyosarcoma
STS	soft tissue sarcomas
TIGIT	T cell immunoreceptor with Ig and ITIM domains
TIM-3	T cell immunoglobulin and mucin domain 3
TKI	tyrosine kinase inhibitor
TMB	tumor mutational burden
TNBC	triple negative breast cancer
TNFα	tumor necrosis factor alpha
T_regs_	regulatory T cells
TP53	tumor protein p53 gene
VEGF	vascular endothelial growth factor
VEGFR	vascular endothelial growth factor receptor
WDLPS	well-differentiated liposarcoma
