# Supplementary material for: From standard therapies to monoclonal antibodies and immune checkpoint inhibitors – an update for reconstructive surgeons on common oncological cases
Source: Front Immunol. 2024 Apr 23;15:1276306. doi: 10.3389/fimmu.2024.1276306 (PMC11074450; doi:10.3389/fimmu.2024.1276306)
Supplement: Supplementary file 1 [file Table_1.docx]

**Supplementary Material 1. Different immunotherapies and their half-life. Guideline recommendations on perioperative halts remain scarce.**

| **Drug Name** | **Drug Class** | **Half-Life** | **Guideline Recommendations on Perioperative Halt** | **Main Target Structure** |
| --- | --- | --- | --- | --- |
| Atezolizumab | ICI | 27d [189] | No | PD-L1 |
| Bevacizumab | mAB | 11-50d [190] | Yes (six to eight weeks prior to surgery; 28 days after surgery) [191] | VEGF |
| Cabozantinib | others | 120 h (terminal t_1/2_) [192] | No | Multiple tyrosine kinases, including MET, VEGFR, and AXL |
| Cetuximab | mAB | 70-100h [193] | No | EGFR |
| Dabrafenib | others | 67h [193] | No | BRAF V600E |
| Ipilimumab | ICI | 15d [194] | No | CTLA-4 |
| Nivolumab | ICI | 25d [194] | No | PD-1 |
| Olaratumab | mAB | 11d [195] | No | PDGFRα |
| Pembrolizumab | ICI | 26d [196] | No | PD-1 |
| Pertucumab | mAB | 18d [197] | No | HER2/neu |
| Relatlimab | mAB | 26.2d [198] | No | LAG-3 |
| Tramefinib | others | 3.9-4.8d [199] | No | MEK1 and MEK |
| Trastuzumab | mAB | 28.5d [200] | No | HER2/neu |
| Verumafenib | others | 50h [201] | No | BRAF V600E |
| 8H79 | mAB | N/A | No | B7-H3 |
